# Supplementary material for: Neuronal CBP-1 is Required for Enhanced Body Muscle Proteostasis in Response to Reduced Translation Downstream of mTOR
Source: Front Biosci (Landmark Ed). Author manuscript; Available in PMC 2024 Sep 19. (PMC11412575; doi:10.31083/j.fbl2907264)
Supplement: Westerns and gels [file NIHMS2017410-supplement-Westerns_and_gels.pdf]

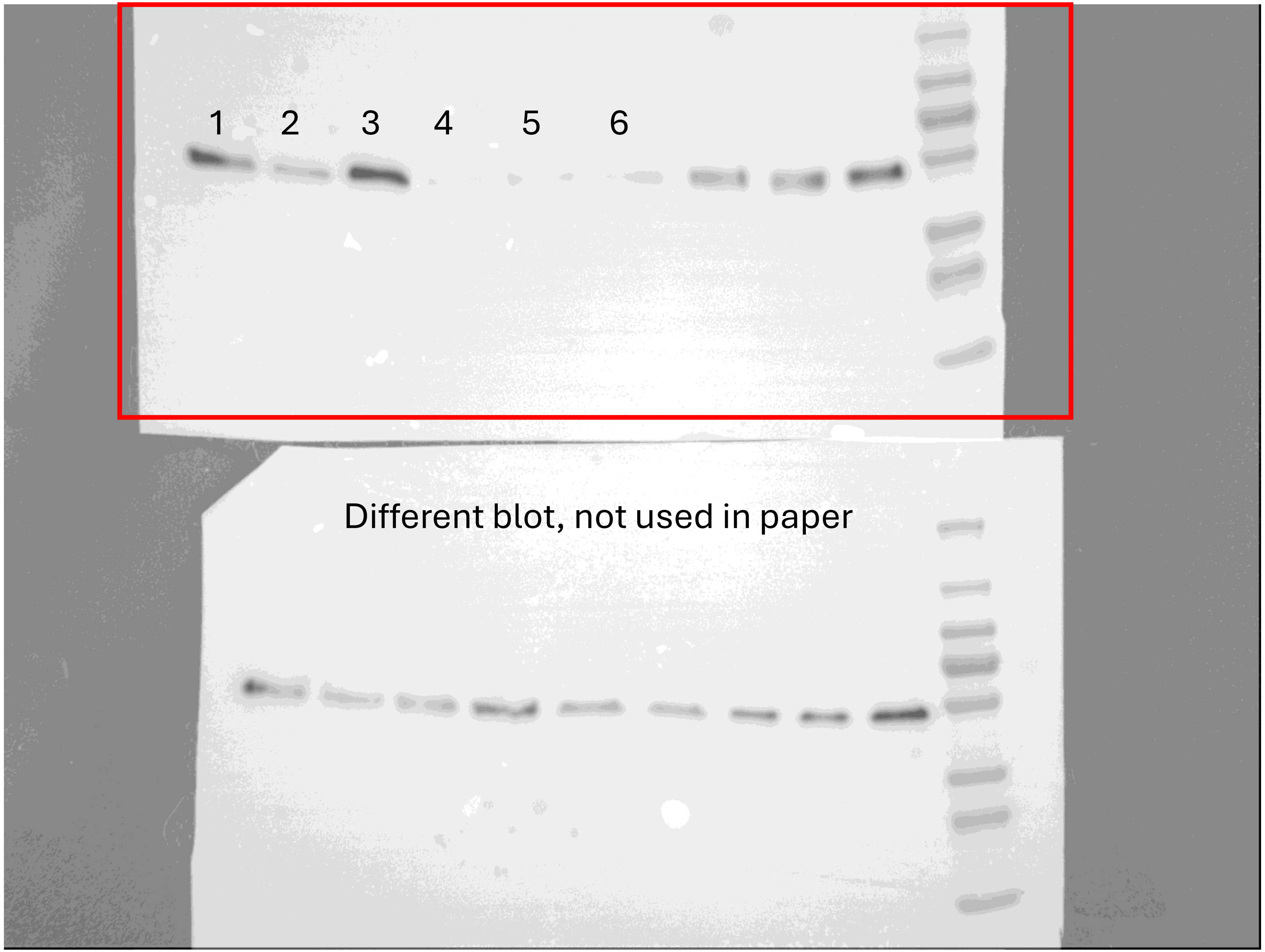

FIGURE 2D
Alpha-synuclein probe blot results

1- ifg1 1

2-ifg-1 2

3- ifg-1 3

4- ctl 1

5- ctl 2

6- ctl 3

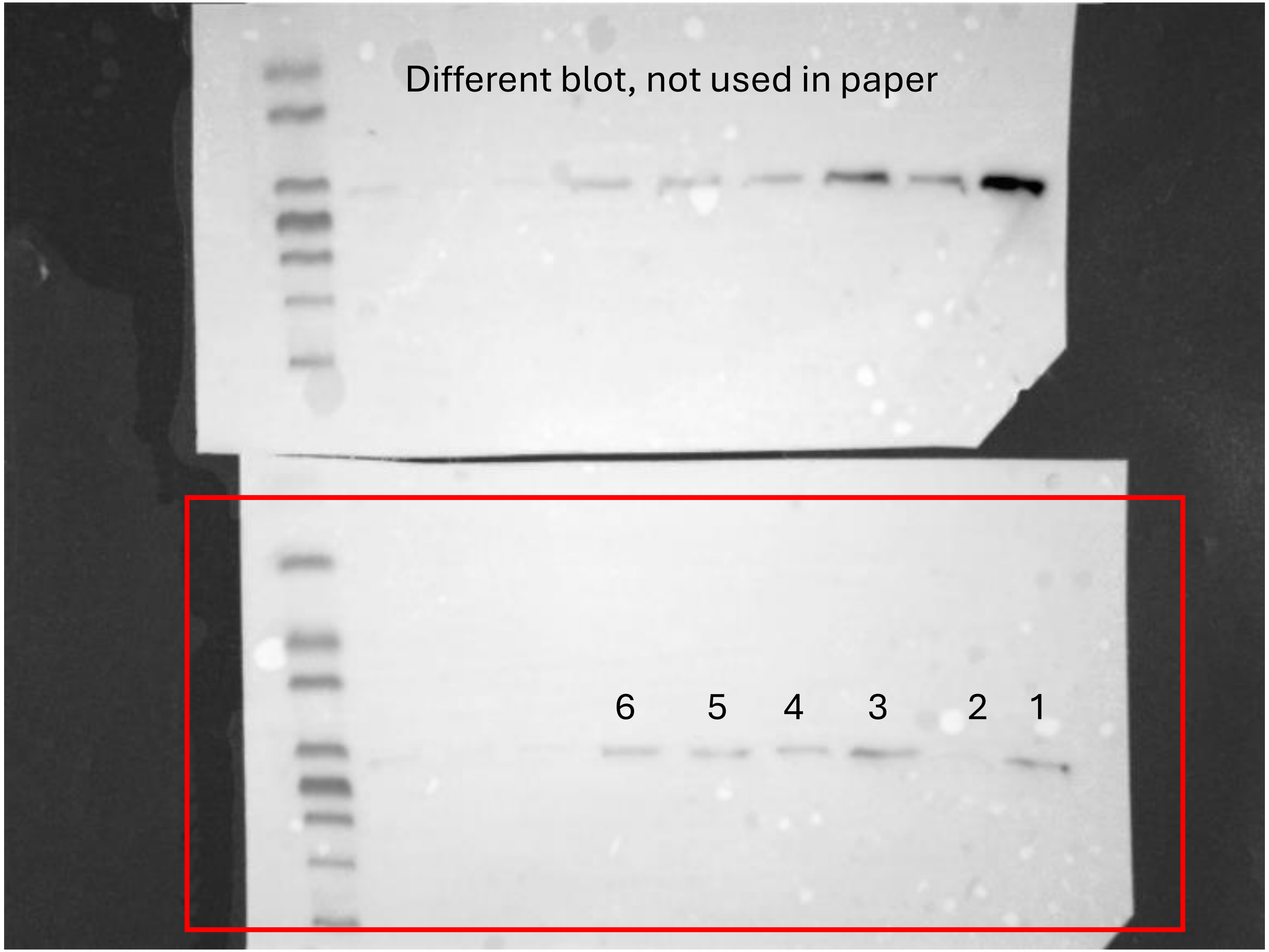

FIGURE 2D

Beta-tubulin probe blot results

1- ifg1 1

2-ifg-1 2

3- ifg-1 3

4- ctl 1

5- ctl 2

6- ctl 3

FIGURE 4B Alpha-synuclein probe blot results

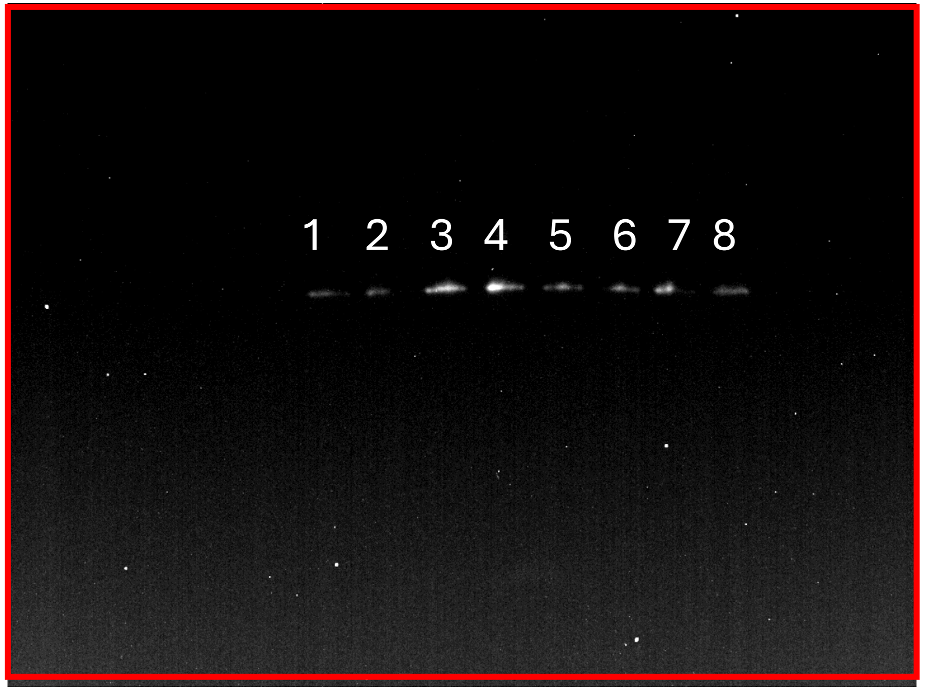

Shown in paper:

1-ctl 1

2-ctl 2

3- ifg-1:ctl 1

4- ifg-1:ctl 2

5-ctl:cbp-1 1

6-ctl:cbp-1 2

7-ifg-1:cbp-1 1

8-ifg-1:cbp-1 2

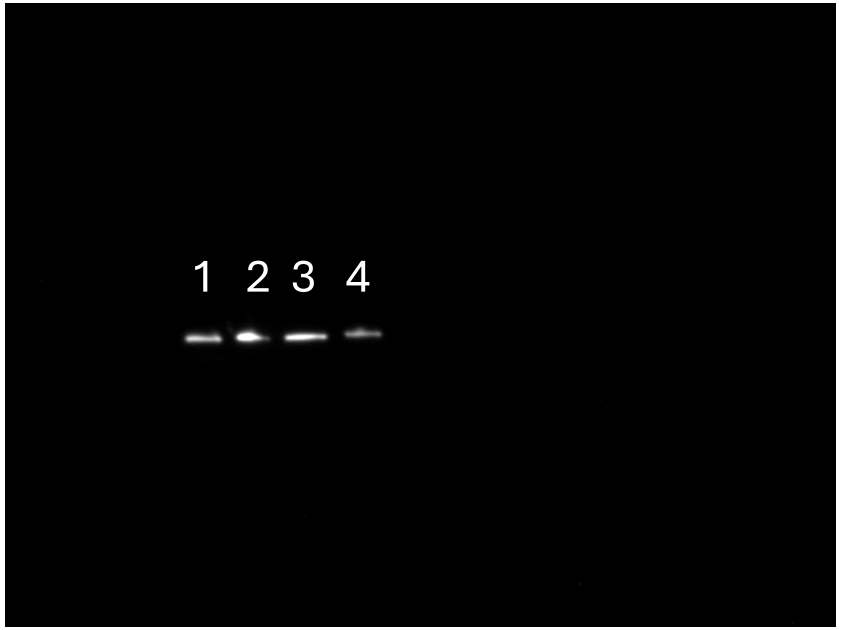

Not shown in paper, but used to quantify 4B:

1-ctl 3

2-ifg-1:ctl 3

3-ctl:cbp-1 3

4-ifg-1:cbp-1 3

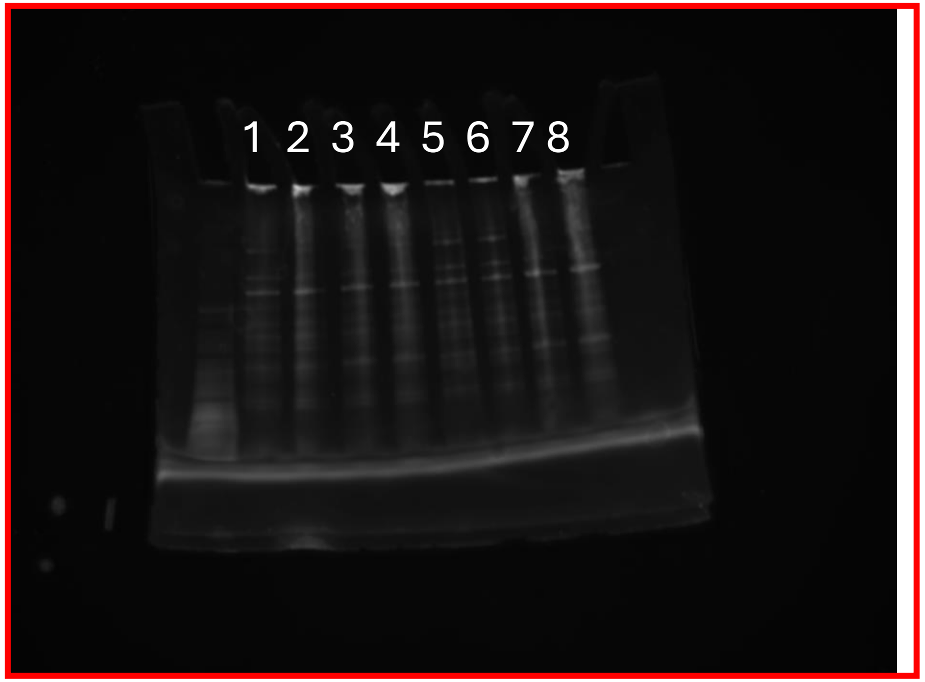

FIGURE 4B Total protein gel results

Shown in paper:

1-ctl 1

2-ctl 2

3- ifg-1:ctl 1

4- ifg-1:ctl 2

5-ctl:cbp-1 1

6-ctl:cbp-1 2

7-ifg-1:cbp-1 1

8-ifg-1:cbp-1 2

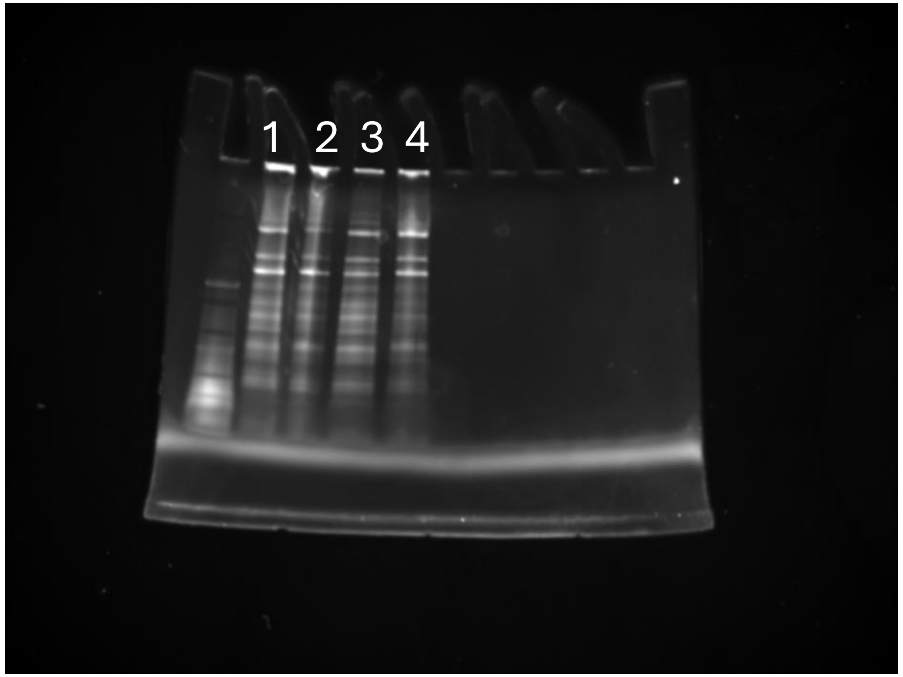

Not shown in paper, but used to quantify 4B:

1-ctl 3

2-ifg-1:ctl 3

3-ctl:cbp-1 3

4-ifg-1:cbp-1 3
